# Left ventricular dyssynchrony in patients with left bundle branch block and patients after myocardial infarction using 3D MR tagging

**DOI:** 10.1186/1532-429X-11-S1-P78

**Published:** 2009-01-28

**Authors:** Robert Manka, Andrea Rutz, Sebastian Kozerke, Peter Boesiger, Jürg Schwitter

**Affiliations:** 1 German Heart Institute Berlin, Berlin, Germany; 2 Institute for Biomedical Engineering, University and ETH, Zürich, Switzerland; 3 Clinic of Cardiology, University Hospital, Zürich, Switzerland

**Keywords:** Myocardial Infarction, Cardiac Resynchronization Therapy, Left Bundle Branch Block, Delay Enhancement, Improve Patient Selection

## Aims

To explore the value of accelerated 3D magnetic resonance (MR) tagging and delayed enhancement (DE) imaging to describe LV dyssynchrony in patients with left bundle branch block (LBBB) and in patients after myocardial infarction (MI).

## Methods

25 patients (60.5 ± 10.4 years) after myocardial infarction, 15 patients (62.9 ± 10.1 years) with left bundle branch block and 15 healthy controls (53.3 ± 9.7 years) underwent 3D tagging [1] and DE imaging at 1.5 T. Spatial resolution was 3.0 × 7.7 × 7.7 mm^3^ with a temporal resolution of 27 ms. Data acquisition was split into three breath-holds with heart position being monitored and corrected for by a navigator technique. The standard deviation (SD) of T_max_ of all segments, the CURE index, and a segmental-based systolic dyssynchrony index (SDI) were calculated as measures of LV dyssynchrony.

## Results

All three parameters detected significantly increased dyssynchrony in patients compared to controls. The standard deviation of T_max_ was significantly higher in patients with MI (78.1 ± 12.6 ms) and with LBBB (73.5 ± 17.0 ms) than in controls (47.2 ± 9.4 ms, p < 0.001 for both). The CURE and SDI showed similar characteristics and detected most dyssynchrony in LBBB-Patients. Bland-Altman plots demonstrate high inter-study and inter-observer reproducibility for CURE and intermediate reproducibility for the SDI and T_max_ SD. Scar mass in the 25 MI patients was 24.2 ± 10.2% of the total LV mass.

## Discussion

3D tagging was successfully applied to provide detailed information on LV-dyssynchrony in patients with LBBB and in patients after myocardial infarction (MI). In combination with delayed enhancement imaging, this approach may show potential to improve patient selection and individual responsiveness for cardiac resynchronization therapy (CRT).
